# Supplementary material for: Association between HTLV-1/2 infection and COVID-19 severity in a migrant Shipibo-Konibo population in Lima, Peru
Source: PLOS Glob Public Health. 2024 Jul 10;4(7):e0003442. doi: 10.1371/journal.pgph.0003442 (PMC11236200; doi:10.1371/journal.pgph.0003442)
Supplement: S1 File — (DOCX) [file pgph.0003442.s001.docx]

**SUPPLEMENTARY MATERIAL**

**Association between HTLV-1/2 infection and COVID-19 severity in a migrant Shipibo-Konibo population in Lima, Peru**

Fátima Avila Dextre^1^, Bryan Morales Álvarez^1^, Paulo Aguirre Castañeda^1^, Isaac Efrain Alva^2^, Giovanni López^3^, Alvaro Schwalb^3,4^, Eduardo Gotuzzo^1,3^

*^1^School of Medicine, Universidad Peruana Cayetano Heredia, Lima, Peru; ^2^School of Public Health, Universidad Peruana Cayetano Heredia, Lima, Peru; ^3^Instituto de Medicina Tropical Alexander von Humboldt, Universidad Peruana Cayetano Heredia, Lima, Peru; ^4^Department of Infectious Disease Epidemiology, London School of Hygiene & Tropical Medicine, London, United Kingdom*

**Supplementary methods**

We used logistic regression to evaluate the association between HTLV-1/2 infection with severe COVID-19, reporting the odds ratio (OR) with their corresponding 95% confidence intervals (CI). The model assumed a binomial distribution of the response variable and employed the logit link function. The model formula is as follows:

$$logit\left( P\left( Y=1 \right) \right)=\beta_{0}+\beta_{1}X_{1}+\beta_{2}X_{2}+. . .+\beta_{n}X_{n}$$

Where the logit function is defined as: $logit\left( P \right)=\log\left( \frac{P}{1-P} \right)$

Y is the response variable, ‘severe COVID-19’, β_0_ is the intercept of the model, β_n_ are the coefficients for each predictor, and X_n_ are the covariates as follows: X_1_: age; X_2_: sex; X_3_: overcrowding (defined as number of rooms divided by number of inhabitants in the household greater than or equal to 3); X_4_: HTVL-1/2 infection (defined as a positive Western-Blot test); X_5_: diabetes mellitus type II; X_6_: asthma; X_7_: anaemia; X_8_: arterial hypertension; and X_9_: dyslipidemia. For secondary and exploratory analyses additional covariates such as overall comorbidities and COVID-19 vaccination were explored.

Variable selection was guided by clinical and epidemiological relevance, supported by theoretical justifications and prior research findings (Garzón-Orjuela et al., 2022; Lima-Martínez et al., 2021; Liu et al., 2021; López-Tiro et al., 2022; Nakazaki et al., 2023; Pepera et al., 2022; Wang et al., 2022; Zheng et al., 2020). No statistical methods or algorithms (stepwise, forward selection, or backward elimination) were used to select the logistic regression covariates. These variables were considered possible confounders in the association between HTLV-1/2 infection and severe COVID-19.

The performance of the model was assessed using the area under the ROC curve, which was 0.68, demonstrating the moderate discriminative ability of the model to correctly classify patient outcomes.

**References**

Garzón-Orjuela, N., Eslava-Schmalbach, J., Gil, F., & Guarnizo-Herreño, C. (2022). Plan de seguro de salud: factor que más contribuye a las desigualdades en la mortalidad por COVID-19 en Colombia. *Revista Panamericana De Salud Publica-Pan American Journal of Public Health*, *46*. https://doi.org/10.26633/rpsp.2022.78

Lima-Martínez, M. M., Carrera Boada, C., Madera-Silva, M. D., Marín, W., & Contreras, M. (2021). COVID-19 y diabetes mellitus: una relación bidireccional. *Clinica e Investigacion En Arteriosclerosis: Publicacion Oficial de La Sociedad Espanola de Arteriosclerosis*, *33*(3), 151–157.

Liu, Y., Pan, Y., Yin, Y., Chen, W., & Li, X. (2021). Association of dyslipidemia with the severity and mortality of coronavirus disease 2019 (COVID-19): a meta-analysis. *Virology Journal*, *18*(1), 157.

López-Tiro, J. J., Contreras-Contreras, E. A., Cruz-Arellanes, N. N., Camargo-Pirrón, M. A., Cabrera-Buendía, E. O., Ramírez-Pérez, G. I., & Vega-Acevedo, G. (2022). [Asthma and COVID-19]. *Revista alergia Mexico* , *69 Suppl 1*, s15–s23.

Nakazaki, J., Gotuzzo, E., Mejía, F., & Montes, M. (2023). SARS-CoV-2 (COVID-19) infection in patients with HTLV-1 infection in Peru - case series. *Journal of Human Virology & Retrovirology*, *10*(1), 15–19.

Pepera, G., Tribali, M.-S., Batalik, L., Petrov, I., & Papathanasiou, J. (2022). Epidemiology, risk factors and prognosis of cardiovascular disease in the Coronavirus Disease 2019 (COVID-19) pandemic era: a systematic review [Review of *Epidemiology, risk factors and prognosis of cardiovascular disease in the Coronavirus Disease 2019 (COVID-19) pandemic era: a systematic review*]. *Reviews in Cardiovascular Medicine*, *23*(1), 28.

Wang, Y., Nan, L., Hu, M., Zhang, R., Hao, Y., Wang, Y., & Yang, H. (2022). Significant association between anemia and higher risk for COVID-19 mortality: A meta-analysis of adjusted effect estimates. *The American Journal of Emergency Medicine*, *58*, 281–285.

Zheng, Z., Peng, F., Xu, B., Zhao, J., Liu, H., Peng, J., Li, Q., Jiang, C., Zhou, Y., Liu, S., Ye, C., Zhang, P., Xing, Y., Guo, H., & Tang, W. (2020). Risk factors of critical & mortal COVID-19 cases: A systematic literature review and meta-analysis. *The Journal of Infection*, *81*(2), e16–e25.

**Table A. Risk factors associated with COVID-19 severity in residents of the Shipibo-Konibo migrant community in Cantagallo, Lima.**

| **Variables** | **Asymptomatic and mild COVID-19 (n=127)** | **Moderate COVID-19 (n=55)** | **Odds ratio (95%CI)** | **Adjusted odds ratio (95%CI)** | **P value** |
| --- | --- | --- | --- | --- | --- |
| Age, years, median (IQR) | 31 (24-43) | 38 (32-47) | 1.03 (1.00-1.06) | 1.03 (0.99-1.05) | 0.068 |
| Male gender | 40 (31.5) | 18 (32.7) | 1.06 (0.53-2.08) | 1.22 (0.58-2.56) | 0.608 |
| Overcrowding | 53 (41.7) | 27 (49.1) | 1.35 (0.71-2.54) | 1.38 (0.69-2.75) | 0.365 |
| HTLV-1/2**^†^** | 20 (15.7) | 7 (12.7) | 0.78 (0.30-1.97) | 0.54 (0.19-1.51) | 0.242 |
| Vaccinated | 43 (33.9) | 30 (54.6) | 2.34 (1.23-4.71) | 2.30 (1.14-4.65) | 0.020 |
| Diabetes mellitus | 8 (6.3) | 6 (10.9) | 1.82 (0.60-5.52) | 2.26 (0.57-8.92) | 0.244 |
| Asthma | 4 (3.1) | 1 (1.8) | 0.57 (0.06-5.21) | 0.17 (0.01-3.62) | 0.254 |
| Anaemia***^‡^*** | 18 (14.3) | 10 (18.5) | 1.36 (0.58-3.18) | 1.56 (0.63-3.86) | 0.341 |
| Arterial hypertension | 1 (0.8) | 4 (7.3) | 9.88 (1.08-90.57) | 9.81 (0.75->99.99) | 0.082 |
| Dyslipidaemia | 1 (0.8) | 1 (1.8) | 2.33 (0.14-38.0) | 0.66 (0.01-43.16) | 0.844 |

*Values are n (%) unless otherwise indicated.* ***^†^****According to HTLV-1/2 WB and ELISA result.* ***^‡^****According to haematocrit values obtained in this study (normal values: males = 41-53%, females = 36-46%). IQR: Interquartile range.*
