# Supplementary material for: Association between HTLV-1/2 infection and COVID-19 severity in a migrant Shipibo-Konibo population in Lima, Peru
Source: PLOS Glob Public Health. 2024 Jul 10;4(7):e0003442. doi: 10.1371/journal.pgph.0003442 (PMC11236200; doi:10.1371/journal.pgph.0003442)
Supplement: S1 Questionnaire — (PDF) [file pgph.0003442.s003.pdf]

Ficha de Recolección de Datos

Proyecto ASOCIACIÓN ENTRE EL IMPACTO DE LA PANDEMIA COVID-19 Y LA INFECCIÓN POR HTLV EN LA POBLACIÓN SHIPIBO-KONIBO MIGRANTE EN LIMA, PERÚ

**INFORMACIÓN GENERAL**	
1. CÓDIGO:	2. DNI:
3. NOMBRES Y APELLIDOS:	
4. PUEBLO INDÍGENA:	
5. LUGAR DE ORIGEN:	
6. EDAD:	7. SEXO : Hombre ___ Mujer ___
8. DIRECCIÓN:	
9. TELÉFONO:	
10. GRADO DE INSTRUCCIÓN:<br>Ninguna ___<br>Primaria ___ (incompleta ___ completa ___)<br>Secundaria ___ (incompleta ___ completa ___)<br>Superior ___ (incompleta ___ completa ___)	
11. OCUPACIÓN: Sí ___ No ___<br>¿Cuál?:<br>¿Su trabajo se vio afectado por la pandemia COVID-19? Sí ___ No ___<br>¿Cómo?:	
12. VIAJES DURANTE LA PANDEMIA COVID-19: Sí ___ No ___<br>Si la respuesta es sí, ¿a qué lugares viajó/por cuánto tiempo?: _________________	
**ANTECEDENTES SOCIODEMOGRÁFICOS**	
13. TIPO Y MATERIAL DE LA VIVIENDA:	
14. NÚMERO DE HABITACIONES/PERSONAS:	
15. CUENTA CON: SISTEMA DE AGUA Sí ___ No ___<br>SISTEMA DE DESAGÜE Sí ___ No ___<br>ÁREAS VENTILADAS Sí ___ No ___	
16. ¿Usted se reúne con sus familiares o amigos para desayunar/almorzar/cenar?<br>Nunca ___ A veces (1 o 2 veces/semana) ___<br>Frecuentemente (>2 veces/semana) ___ Siempre ___	
**ANTECEDENTES PATOLÓGICOS**	
17. ENFERMEDADES: Sí ___ No ___, si la respuesta es sí, cuáles?<br>Diabetes tipo 2 ___ HTA ___ Obesidad ___ Asma ___<br>Dislipidemia ___ Anemia ___ Cáncer (tipo) ___________________________<br>Tuberculosis pulmonar (tipo/tto/tiempo de tto/control) ___________________________<br>Infección por dengue ___ VIH ___ Micosis cutánea ___ Onicomicosis ___	

Infección por *Strongyloides* ___ Acarosis ___ Sarna o “rasca rasca” ___
Paracoccidioidomicosis ___ Histoplasmosis ___ Leishmaniasis ___
Uveítis o lesión ocular ___ Tiroiditis ___ Hepatitis ___
Fibrosis pulmonar ___ Otros: ________________________________

18. HOSPITALIZACIONES PREVIAS: Sí ___ No ___ Especificar: _______________

19. INTERVENCIONES QUIRÚRGICAS: Sí ___ No ___ Especificar: _______________

20. MEDICINAS DE USO FRECUENTE: Sí ___ No ___
Especificar: ____

21. REACCIONES ADVERSAS A MEDICAMENTOS Y ALERGIAS: Sí ___ No ___
Especificar: ____

22. VACUNAS/INMUNIZACIONES: Sí ___ No ___ Especificar:
BCG ___ Fiebre amarilla ___ Contra neumonía (neumococo) ___
Difteria/tétanos ___ Hepatitis B ___ Contra la influenza ___

23. ELIMINACIÓN DE PARÁSITOS: Sí ___ No ___
¿Recibió antiparasitario? Sí ___ No ___ Especificar: ______________

24. ACCIDENTES Y SECUELAS: Sí ___ No ___ Especificar: ______________

25. TRANSFUSIONES DE SANGRE Y/O DERIVADOS: Sí ___ No ___
Especificar: ____

26. HÁBITOS NOCIVOS:
Alcohol: ___ Tabaco (pqte/año): _______________
Uso de drogas: Sí ___ No ___ Si la respuesta fue sí, cuáles?
Cocaína ___ Marihuana ___ Ayahuasca ___ Otros: _______

27. ANTECEDENTES GINECO-OBSTÉTRICOS:
FUR: _______ G PARA: ________ Aborto/Natimuerto previos: _______________
Último PAP: ______

INFORMACIÓN RELACIONADA A COVID-19:

32. Diagnóstico de COVID-19 **anteriormente a este estudio**: Sí ___ No ___
Si la respuesta es sí, ¿qué tipo de prueba diagnóstica le hicieron?:
prueba rápida ___ (¿cuál fue el resultado? IgM+ --- IgG+ --- IgG/IgM+)
prueba molecular ___
Fecha de la prueba: ____________
En caso de mujer, ¿estaba embarazada al momento del diagnóstico?: Sí ___ No ___

33. Condición clínica al momento de la toma de prueba:
- Asintomático: ___
- Sintomático: ___
  Tos seca: ___ Fiebre: ___ Disnea: ___ Cefalea: ___ Diarrea: ___
  Alteración del olfato: ___ Alteración del gusto: ___ Otros: _____________

34. Medicamentos o medicina natural usados para **tratar** la COVID-19: Sí ___ No ___
- Medicina natural: ___ (especificar cuál: ____________________________)
- Ivermectina: ___

- Hidroxicloroquina: ___
- Azitromicina: ___
- Corticoides (prednisona o dexametasona): ___
- Paracetamol: ___
- Otros: ________________________________

Especificar (dosis/frecuencia): ____
____

35. Medicamentos o medicina natural usados para **prevenir** la COVID-19: Sí ___ No ___
    - Medicina natural: ___ (especificar cuál: ______________________________)
    - Ivermectina: ___
    - Hidroxicloroquina: ___
    - Azitromicina: ___
    - Corticoides (prednisona o dexametasona): ___
    - Paracetamol: ___
    - Otros: ________________________________

    Especificar (dosis/frecuencia): ____
    ____

36. ¿Solicitó atención de salud durante la pandemia? Sí ___ No ___
    Si la respuesta fue sí, ¿a quién? línea telefónica del MINSA ___ Centro de Salud ___
    Otro: _____________________________________
    ¿recibió dicha atención? Sí ___ No ___

37. Hospitalización por COVID-19: Sí ___ No ___
    Si la respuesta fue sí, ¿estuvo en UCI? Sí ___ No ___

38. Actualmente presenta alguna afección (secuelas) como consecuencia del COVID-19?
    Sí ___ No ___ Si la respuesta es sí, ¿cuáles?
    Alteración del sueño ___ Alteración del ánimo ___ Fatiga ___ Mareos ___
    Alteración del olfato ___ Dolor torácico ___ Disnea ___ Cefalea ___
    Alteración del gusto ___ Artralgias ___ Otros: _____________________

39. Familiares con COVID-19: Sí ___ No ___
    ¿Alguien en casa ha tenido COVID-19? Sí ___ No ___
    ¿Cuántos? ___ Parentesco: ___________________________________
    ¿Fue hospitalizado por COVID-19? Sí ___ No ___
    ¿Falleció con COVID-19 en 2020-2021?: Sí ___ No ___
    ¿Cuántos? ___ Parentesco: ______________________________
    Número de contacto del familiar con COVID-19 en caso no viva en casa:
    ____
    ____

INFORMACIÓN RELACIONADA A HTLV-1/2:

40. Diagnóstico de HTLV-1/2 **anteriormente a este estudio**: Sí ___ No ___
    (Si la respuesta es **No** pasar a la pregunta 44)
    Fecha de la prueba **anteriormente a este estudio**: ___________
    En caso de mujer, ¿estaba embarazada al momento del diagnóstico?: Sí ___ No ___

41. Condición clínica al momento de la toma de prueba **anteriormente a este estudio**:
    - Asintomático: ___
    - Sintomático: ___

42. ¿Tiene alguna complicación debido al HTLV-1/2?: Sí ___ No ___
Si la respuesta es sí, ¿Cuáles?
Mielopatía/paraparesia espástica tropical - PET ___
Leucemia/linfoma de células T del adulto - LLTA ___
Tumores o cáncer (gástrico, cuello uterino, etc) ___
¿Cuál? ____
Uveítis o lesión ocular ___ Tiroiditis ___
Fibrosis pulmonar ___ Hepatitis ___
Infección por Strongyloides ___ TBC ___ Acarosis ___
Micosis cutánea ___ Onicomicosis ___ Histoplasmosis ___
Paracoccidioidomicosis ___ Leishmaniasis ___ Sarna o "rasca rasca" ___
Otros: ______________________________

43. Actualmente, le realizan algún seguimiento?: Sí ___ No ___
Si la respuesta es sí, ¿En cuál establecimiento de salud?: ________________________

44. Familiares diagnosticados con HTLV-1/2: Sí ___ No ___
Si la respuesta es sí, ¿Quiénes? ____
Número de contacto del familiar: ____

Resultado de la prueba para diagnosticar HTLV-1/2: _____________
Fecha de la prueba: __________________
En caso de mujer, ¿reconoce que está embarazada al momento del diagnóstico?
Sí ___ No __
