# Supplementary material for: Association between HTLV-1/2 infection and COVID-19 severity in a migrant Shipibo-Konibo population in Lima, Peru
Source: PLOS Glob Public Health. 2024 Jul 10;4(7):e0003442. doi: 10.1371/journal.pgph.0003442 (PMC11236200; doi:10.1371/journal.pgph.0003442)
Supplement: Attachment — Submitted filename: Response to Reviewers.docx [file pgph.0003442.s005.docx]

**Response to reviewer comments**

We thank the reviewers for their constructive review of our work. We have revised the manuscript accordingly. Please find the response to the comments made below:

1. **Explain the criteria for identifying mild, moderate, and severe cases.**

We thank the reviewer for this comment. We have added a table which list the criteria needed to be classified as mild, moderate, and severe case of COVID-19. This was the same criteria employed nationally during the pandemic.

2. **Perform a separate analysis for patients without comorbidities.**

As requested, we performed a separate analysis that only included participants without comorbidities, as those could have affected the severity of disease. This is shown in Table 7 where we observe similar results to the full analysis, i.e., only age is a statistically significant risk factor.

3. **Explain whether the HTLV positive patients had ATL or HAM/TSP.**

We acknowledge that this should have been mentioned in the text. We have included a sentence mentioning that no participant suffered from ATL or HAM/TSP (Line 140-142: “*None of the participants with WB HTLV-1/2 positive results presented any comorbidities associated with HTLV-1/2 infection, such as tropical spastic paraparesis and adult T-cell leukaemia/lymphoma*.”)

Please let us know if we can provide any additional information. We look forward to hearing from you at your earliest convenience.

Warm wishes,

Fátima Ávila, for the authors
