# Supplementary material for: Association between HTLV-1/2 infection and COVID-19 severity in a migrant Shipibo-Konibo population in Lima, Peru
Source: PLOS Glob Public Health. 2024 Jul 10;4(7):e0003442. doi: 10.1371/journal.pgph.0003442 (PMC11236200; doi:10.1371/journal.pgph.0003442)
Supplement: Attachment — Submitted filename: Response to Reviewers.docx [file pgph.0003442.s006.docx]

**Response to reviewer comments**

We thank the reviewers for their constructive review of our work. We have revised the manuscript accordingly. Please find the response to the comments made below:

**Reviewer 2:**

1. **Line 261, The study has clear limitations as the authors already mentioned "...which adds uncertainty to whether the test was positive due to SARS-CoV-2 infection, false positivity, or vaccination". The study should control vaccination status, especially the authors cannot make sure whether positive test was due to natural infection or vaccination. Therefore, please also show the regression results after separating vaccinated and unvaccinated.**

We thank the reviewer for this comment. We agree and have added vaccination status to the analysis with respect to COVID-19 (Table 4). We also performed a sensitivity analysis where we evaluated the impact of vaccination on COVID-19 severity, finding a significant but inverse association to what is expected from vaccination. We believe this to be a spurious result and thus have opted to not present the table from this analysis in the main body of the manuscript but rather within the Supplementary Material. However, we did note this in the manuscript: *“Additionally, another sensitivity analysis was conducted where the impact of SARS-CoV-2 vaccination on the severity of COVID-19 was evaluated showing a significant but inverse association than expected (aOR: 2.30; 95%CI: 1.14-4.65; p=0.020) (Supplementary Material).”* (Line 196-199)

2. **Show the proportion of participants among asymptomatic, mild, and moderate for vaccinated and unvaccinated as Table 4.**

As above, we followed the reviewer’s comment and added vaccination status to the analysis in Table 4.

3. **Line 113, "We used logistic regression to evaluate the association between HTLV-1/2 infection with severe COVID-19 and reported the odds ratio (OR)", authors should give more detailed descriptions of logistic model. Did authors performed variable selection or model comparison to determine an optimal set of variables?**

We have now explicitly described the methods used to determine the set of variables: *“Risk factors for the multivariable model were chosen based on clinical and epidemiological relevance.”* (Line 118-119).

4. **The variable overcrowding in the model was not described and explained.**

We thank the reviewer for noting this. We have now described the variable in the Methods section: *“We defined overcrowding for participants as a condition where the number of rooms divided by number of inhabitants in the household was greater than or equal to 3.”* (Line 81-83)

5. **The difference of age distribution between HTLV(+) and HTLV(-) is limitation. Please discuss it.**

We have discussed the age distribution according to HTLV status in the limitations of the study: *“The disparity in age distribution between the HTLV-1/2 positive and negative groups could pose a limitation because the more severe COVID-19 outcomes observed in the HTLV-1/2 positive group might be attributed to their older age rather than not their HTLV-1/2 status.”* (Line 276-279)

Please let us know if we can provide any additional information. We look forward to hearing from you at your earliest convenience.

Warm wishes,

Fátima Avila, for the authors
