# Supplementary material for: Association between HTLV-1/2 infection and COVID-19 severity in a migrant Shipibo-Konibo population in Lima, Peru
Source: PLOS Glob Public Health. 2024 Jul 10;4(7):e0003442. doi: 10.1371/journal.pgph.0003442 (PMC11236200; doi:10.1371/journal.pgph.0003442)
Supplement: Attachment — Submitted filename: Response to Reviewers.docx [file pgph.0003442.s007.docx]

**Response to reviewer comments**

We thank the reviewer for their constructive review of our work. We have revised the manuscript accordingly. Please find the response to the comments made below:

**Reviewer 2:**

1. **Please make sure all your data or files are fully available.**

Apologies that the files were not available for you to review. We made a mistake while uploading the files and categorizing them as ‘Others’ rather than ‘Supporting Information’. We have made sure that all relevant supplementary files have been uploaded correctly in this new revision round.

2. **Regarding the methods used to select variables for the model: I appreciated that you have added more words to describe your model, but this is still not clear enough. Please add your probability distribution, link function and regression model formula including all covariants. Did authors perform variable selection or model comparison to determine an optimal set of variables? How to know model fit and how to decide which variables should be included or excluded? Please address these questions otherwise you may get different results.**

Thanks for the clarification, we have expanded and detailed the methods used for the logistic regression in the manuscript including the probability distribution, link function, as well as providing the regression model formula in the Supplementary Material. There we also provide references that informed our decision of what covariates to include in the model. For the manuscript, this is written as: “*We used logistic regression to evaluate the association between HTLV-1/2 infection with severe COVID-19, reporting the odds ratio (OR) with their corresponding 95% confidence intervals (CI). The model assumed a binomial distribution of the response variable and employed the logit link function. Details on the model formula are provided in the Supplementary Material. Variable selection was guided by clinical and epidemiological relevance, supported by theoretical justifications and prior research findings. The performance of the model was assessed using the area under the ROC curve, which was 0.68, demonstrating the moderate discriminative ability of the model to correctly classify patient outcomes.*”

Please let us know if we can provide any additional information. We look forward to hearing from you at your earliest convenience.

Warm wishes,

Fátima Ávila, for the authors
