# Supplementary material for: Association between HTLV-1/2 infection and COVID-19 severity in a migrant Shipibo-Konibo population in Lima, Peru
Source: PLOS Glob Public Health. 2024 Jul 10;4(7):e0003442. doi: 10.1371/journal.pgph.0003442 (PMC11236200; doi:10.1371/journal.pgph.0003442)
Supplement: Attachment — Submitted filename: Response to Reviewers.docx [file pgph.0003442.s008.docx]

**Response to journal and reviewer comments**

We thank the journal and the reviewer for their constructive review of our work. We have revised the manuscript accordingly. Please find the responses below:

**Journal:**

1. **Please include a complete copy of PLOS’ questionnaire on inclusivity in global research as Supporting Information.**

We have uploaded a complete copy of PLOS’ questionnaire on inclusivity in global research as Supporting Information. Of note, this was also available in our previous submission.

2. **Please review your reference list to ensure that it is complete and correct. If you have cited papers that have been retracted, please include the rationale for doing so in the manuscript text or remove these references and replace them with relevant current references.**

We have fully reviewed the reference list ensuring that it is complete and correct. None of the cited papers have been retracted.

3. **The reviewer feels that the details of the statistical analysis still need to be revised for the research to be reproducible. Please be more specific when describing the analysis methodology.**

We have provided additional details in response to the reviewer.

**Reviewer 2:**

1. **May I suggest you to move your main description of the model in the supplementary information into the methods section in the main text.**

Thanks for the suggestion. After careful consideration, we believe it is not necessary to include all the methodology description text from the Supplementary Material (which also includes the formula and a basic explanation of the logistic regression model with logit link function). This detailed text was a very welcome suggestion that has greatly improved the description of the methods; however, we think that most readers can be informed with the shortened description in the methods.

2. **Could you specify how to access your source codes?**

Thanks for letting us know. We are not sure what you mean with source code. If you are referring to the study database (including the data dictionary), this is available as a link in the PDF generated from the submission. If you are referring to the Stata do file to generate the analysis, we can make this available if necessary but going back to the previous point of the analysis description in Supplementary Material, the results can be generated in Stata or R with the study database and the inclusion of the covariates as described.

3. **About your variable selection, please be more specific to describe the approach. For example, what was the criteria and what variables were removed. I think this part should also be described in the main text to help readers to understand easily.**

Thanks for raising this again, it seems we were not able to accurately describe the variable selection. Variable selection for the model was exclusively guided by clinical and epidemiological relevance, i.e. as researchers and clinicians we were aware of possible confounders in the association between HTLV-1/2 and severe COVID-19 and chose those variables. Thus, no statistical methods or algorithms (stepwise, forward selection, or backward elimination) were used to select the logistic regression covariates. This has now been expanded in both Methods and Supplementary material.

Please let us know if we can provide any additional information. We look forward to hearing from you at your earliest convenience.

Warm wishes,

Fátima Ávila, for the authors
